# Rock phosphate solubilization by abiotic and fungal‐produced oxalic acid: reaction parameters and bioleaching potential

**DOI:** 10.1111/1751-7915.13792

**Published:** 2021-03-12

**Authors:** Gilberto de Oliveira Mendes, Thomas Dyer, Laszlo Csetenyi, Geoffrey Michael Gadd

**Affiliations:** ^1^ Laboratório de Microbiologia e Fitopatologia Instituto de Ciências Agrárias Universidade Federal de Uberlândia Rod. LMG‐746, km 1, Bloco 1A‐MC, Sala 315 Monte Carmelo MG 38500‐000 Brazil; ^2^ Geomicrobiology Group School of Life Sciences University of Dundee Dundee DD1 5EH UK; ^3^ Concrete Technology Group Department of Civil Engineering University of Dundee Dundee DD1 4HN UK; ^4^ State Key Laboratory of Heavy Oil Processing Beijing Key Laboratory of Oil and Gas Pollution Control Department of Environmental Science and Engineering College of Chemical Engineering and Environment China University of Petroleum 18 Fuxue Road Changping District Beijing 102249 China

## Abstract

Oxalic acid‐producing fungi play an important role in biogeochemical transformations of rocks and minerals and possess biotechnological potential for extraction of valuable elements from primary or waste ores and other solid matrices. This research investigates the extraction of phosphate from rock phosphate (RP) by oxalic acid. Reaction parameters were derived using pure oxalic acid solutions to solubilize RP. It was found that the oxalic acid concentration was the main factor driving reaction kinetics. Excess oxalic acid could retard the reaction due to calcium oxalate encrustation on RP surfaces. However, complete P extraction was reached at stoichiometric proportions of apatite and oxalic acid. This reaction reached completion after 168 h, although most of the P (up to 75%) was released in less than 1 h. Most of the Ca released from the apatite formed sparingly soluble calcium oxalate minerals, with a predominance of whewellite over weddellite. Bioleaching of RP employing biomass‐free spent culture filtrates containing oxalic acid (100 mM) produced by *Aspergillus niger* extracted ~ 74% of the P contained in the RP. These findings contribute to a better understanding of the reaction between apatite and oxalic acid and provide insights for potential applications of this process for biotechnological production of phosphate fertilizer.

## Graphical abstract

This research investigates the extraction of phosphate from rock phosphate by abiotic and fungal‐produced oxalic acid. Complete P extraction was achieved at stoichiometric proportions of apatite and oxalic acid, with up to 75% P released in less than 1 h. Most of the Ca released from the apatite formed sparingly‐soluble calcium oxalate minerals.

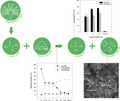

## Introduction

Global food demand is increasing rapidly, with some forecasts indicating the need to double agricultural output from 2005 to 2050 (Alexandratos and Bruinsma, 2012). To achieve this, agricultural intensification on existing low‐yielding croplands is a reasonable means to increase productivity with a low environmental impact, minimizing land clearance and greenhouse gas emissions (Tilman *et al.,* 2011; Withers *et al.,* 2018). One of the main components required to increase crop yields is fertilization, and this will probably result in increasing fertilizer consumption from 166 Mt in 2005 to 263 Mt in 2050 (Alexandratos and Bruinsma, 2012). Among the important nutrient elements, phosphorus (P) occupies the second position in the volume of fertilizer application in the world, after nitrogen (FAO, faostat.fao.org). However, unlike nitrogen‐containing fertilizers which are derived from atmospheric N_2_ through biological or industrial fixation, phosphate fertilizers are obtained mainly from non‐renewable mineral deposits, primarily rock phosphates (RPs). The depletion of RP reserves is concerning and the subject of a vigorous debate since there is a consensus that sustainable P use is paramount for global food security (Cordell *et al.,* 2009; Schröder *et al.,* 2010; Cordell and White, 2011; Withers *et al.,* 2015).

RPs are mainly composed of apatite [Ca_5_(PO_4_)_3_(F,OH,Cl)], a sparingly soluble phosphate mineral. Therefore, RPs cannot meet the P demand of many crops in the short term due to slow phosphate release (Rajan *et al.,* 1996). Because of this, most of the mined RP is converted to highly soluble phosphate fertilizers by treatment with strong acids, mainly sulfuric acid (Hatfield, 1964; Kongshaug *et al.,* 2000; Gilmour, 2014). Sulfuric acid is second only to the RP as constituting the highest production cost in this process (Gilmour, 2014). Sulfuric acid is produced from elemental sulfur, which represents 96% of the production cost, and is mostly obtained from the oil and natural gas industry as a by‐product (Gilmour, 2014). Therefore, the costs of RP processing with sulfuric acid, and thus the price of phosphate fertilizers, are strongly influenced by elemental sulfur demand. This means that processing low‐grade ores is uneconomical due to the required high sulfuric acid consumption (Santos *et al.,* 2002). Moreover, high‐grade RPs are becoming scarcer and more expensive to mine, process and transport, since world reserves are unevenly distributed (Cordell *et al.,* 2009; Schröder *et al.,* 2010; Cordell and White, 2014).

Previous research has highlighted the potential of oxalic acid for RP solubilization (Kpomblekou‐A and Tabatabai, 1994, 2003; Mendes *et al*., 2020a,2020b). Oxalic acid is able to extract 100% of P contained in different RPs from various origins, and is more efficient than sulfuric acid, releasing more P per mol of acid applied (Mendes *et al.,* 2020b). Thus, oxalic acid appears to be a promising alternative for production of phosphate fertilizers. Currently, most of the oxalic acid marketed is produced by a chemical route based on carbohydrate oxidation with sulfuric and nitric acids (Riemenschneider and Tanifuji, 2011). Thus, the substitution of sulfuric acid by chemically synthesized oxalic acid would be uneconomical and still dependent on the supply of sulfuric acid (Mendes *et al.,* 2020b). On the other hand, microbially produced oxalic acid represents a promising alternative for RP bioprocessing, offering an efficient, low‐cost and environmentally friendly method for P extraction (Liang and Gadd, 2017; Mendes *et al.,* 2020b).

Oxalic acid is produced by many and various fungi, including wood‐rotting basidiomycetes, mycorrhizas, phytopathogens and saprotrophs (Dutton *et al.,* 1993; Dutton and Evans, 1996; Sayer and Gadd, 1997; Shimada *et al.,* 1997; Gadd, 1999, 2010, 2017b; Magnuson and Lasure, 2004; Gadd *et al.,* 2014; Mendes *et al.,* 2020a). High‐yield fungal oxalic acid production using renewable carbon sources is easily achieved (Strasser *et al.,* 1994). Moreover, oxalic acid has been reported as an important agent in bioleaching systems for solubilizing metals from low‐grade ores and industrial wastes and by‐products (Strasser *et al.,* 1994; Gadd *et al.,* 2014; Vakilchap *et al.,* 2016; Gadd, 2017a; Liang and Gadd, 2017; Suyamud *et al.,* 2020). Therefore, the aim of this research was to investigate RP solubilization with fungal‐derived oxalic acid to understand the main factors controlling the process and, thus, define the conditions that maximize P extraction in a bioleaching context.

## Results

### RP characterization

The RP sample exhibited a P content of 24.08% expressed as P_2_O_5_ (Table 1). It should be noted that the RP was not subjected to any beneficiation steps and therefore a significant content of contaminants like Al and Fe was also present (Table 1). Mineralogical characterization using Rietveld refinement techniques applied to powder XRD data revealed the RP sample contained apatite (69.3%), goethite (11.1%), dickite (8.5%), quartz (4.9%), muscovite (4.6%) and albite (1.6%).

**Table 1:** Chemical composition of the rock phosphate as determined by X‐ray fluorescence spectrometry. Rock phosphate source: Pratápolis, MG, Brazil.

Component	Content	
P_2_O_5_	24.08	%
CaO	29.39	%
F	1.35	%
Fe_2_O_3_	7.12	%
Al_2_O_3_	8.35	%
MgO	0.78	%
SiO_2_	18.76	%
K_2_O	1.21	%
MnO	0.71	%
Na_2_O	0.16	%
As	88.6	mg kg^−1^
Ba	862.9	mg kg^−1^
Cl	75.7	mg kg^−1^
Co	157.9	mg kg^−1^
Cr	223.5	mg kg^−1^
Cu	56.2	mg kg^−1^
Ni	272.2	mg kg^−1^
Pb	65.6	mg kg^−1^
Rb	32	mg kg^−1^
SO_3_	201.1	mg kg^−1^
Sr	296.2	mg kg^−1^
TiO_2_	3500	mg kg^−1^
Y	96.9	mg kg^−1^
Zn	383.3	mg kg^−1^
Zr	819.5	mg kg^−1^

### Reaction parameters of the RP solubilization with oxalic acid

**Fig. 1:**
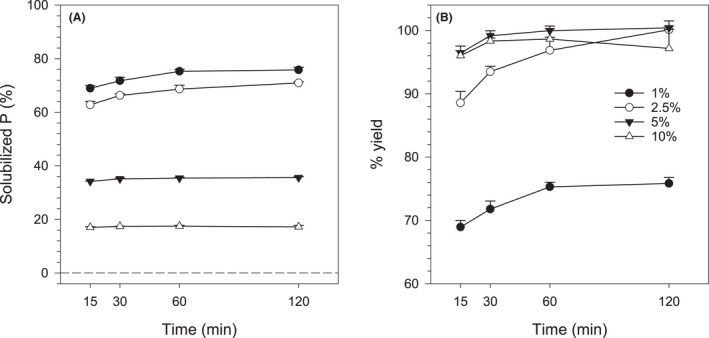
Rock phosphate (RP) solubilization by oxalic acid (100 mM) as a function of the proportion of solids (% RP) and the reaction time. (A) Solubilized P (% of total P added). (B) Percentage yield of the reaction. The dashed line represents the mean of controls without oxalic acid. P was not detected in controls with oxalic acid only. Error bars denote the standard deviation (*n* = 3).

The reaction between oxalic acid and RP was rapid, with most of the P being solubilized over the first hour, reaching up to 75% release (Fig. 1A). After that, little incremental increases in Pi concentration were detected. The proportion of solids affected the amount of P extracted from the RP by oxalic acid in that the lower the amount of RP added, the higher the percentage of solubilized P (Fig. 1A). Conversely, the reaction yield was greatest for the higher proportions of solids (2.5%, 5% and 10%), reaching 100% of the theoretical yield (Fig. 1B). Therefore, over short reaction times only limiting concentrations of oxalic acid reached 100% of the theoretical yield of solubilized P.

**Fig. 2:**
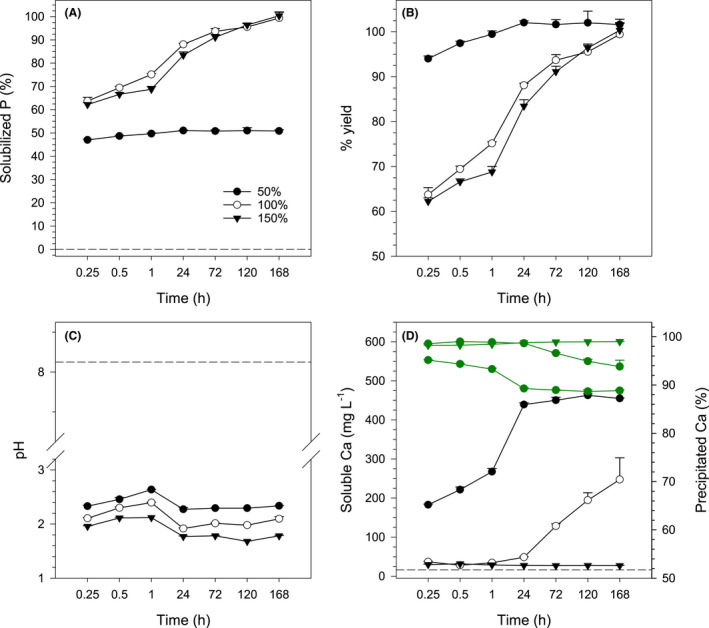
Rock phosphate solubilization as a function of oxalic acid dose (50, 100 and 150% of the required dose) and reaction time. (A) Solubilized P (% of total P added). (B) Percentage yield of the reaction. (C) Equilibrium pH measured after the reaction. (D) Soluble Ca in the reaction medium (black) and percentage of Ca precipitated by oxalate (green) estimated by the difference between the theoretical and measured amount of Ca in the reaction medium. Dashed lines represent the mean of controls without oxalic acid. P or Ca was not detected in controls with oxalic acid only. Error bars denote standard deviation (*n* = 3).

On increasing the reaction time, solubilization was complete at 168 h for concentrations of oxalic at 100% or 150% of the required dose to extract all the P contained in the RP (Fig. 2A). At 50% of the required dose of oxalic acid, the reaction reached the maximum yield in less than 24 h, while at 100% and 150% of the required dose it took 168 h (Fig. 2B).

For all oxalic acid concentrations, the pH of the reaction medium increased until 1 h, and then decreased until 24 h and after that varied slightly (Fig. 2C). Over the time frame evaluated, the higher oxalic acid dose resulted in a lower medium pH.

Most of the Ca released from apatite was rapidly precipitated by oxalate (Fig. 2D). Lower Ca precipitation was observed in the reaction with limiting oxalic acid (50% of the required dose). When 100% of the required dose was applied, more than 98% of the Ca released in the first 24 h was precipitated. With excess oxalic acid, the amount of Ca precipitated remained stable with time, reaching nearly 99% complexation.

### Mineralogical and morphological transformations of RP by oxalic acid

**Fig. 3:**
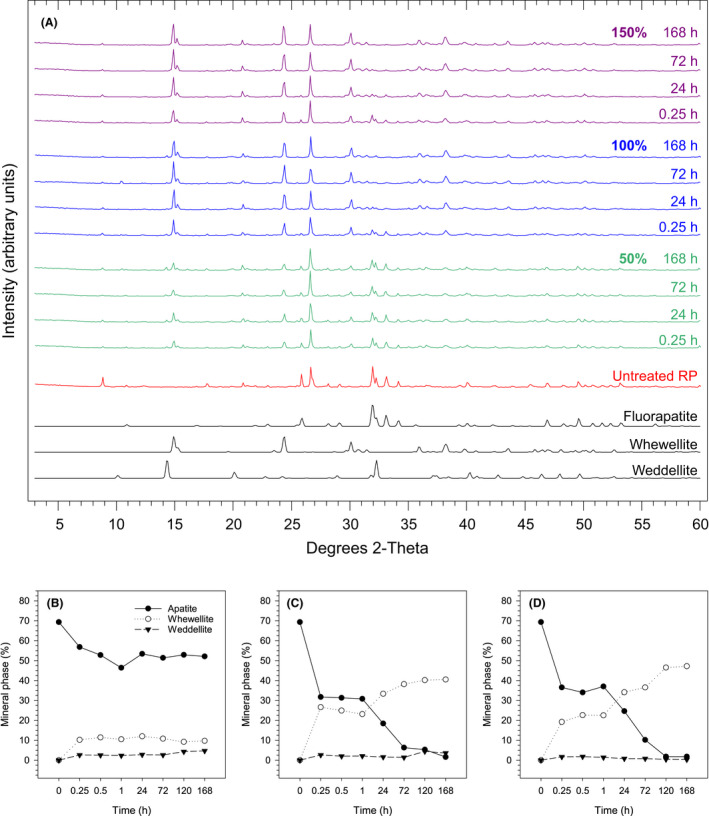
Changes in the mineralogical composition of rock phosphate (RP) during solubilization with oxalic acid at 50%, 100% and 150% of the required dose. (A) Powder XRD patterns of sediments at different reaction times and oxalic acid doses compared to untreated RP and standard patterns of fluorapatite (ICSD 9444), whewellite (ICSD 434201) and weddellite (ICSD 192659). Reaction times similar to those adjacent were omitted for image clarity. Quantitative analysis of the sediments during reaction with oxalic acid at (B) 50, (C) 100 and (D) 150% of the required dose. Mineral phase percentages were estimated using Rietveld refinement techniques applied to powder XRD patterns. Time zero corresponds to the untreated RP. Only mineral phases related to the solubilization reaction are presented.

Data from powder XRD analysis showed that while the apatite was consumed in the reaction, calcium oxalate minerals were formed, with a predominance of whewellite (CaC_2_O_4_·H_2_O) over weddellite (CaC_2_O_4_·2H_2_O) (Fig. 3). When non‐limiting doses of oxalic acid were applied, virtually all the apatite was consumed and the whewellite accounted for about 40% of the mineral phases at the end of the reaction (Fig. 3B,C).

**Fig. 4:**
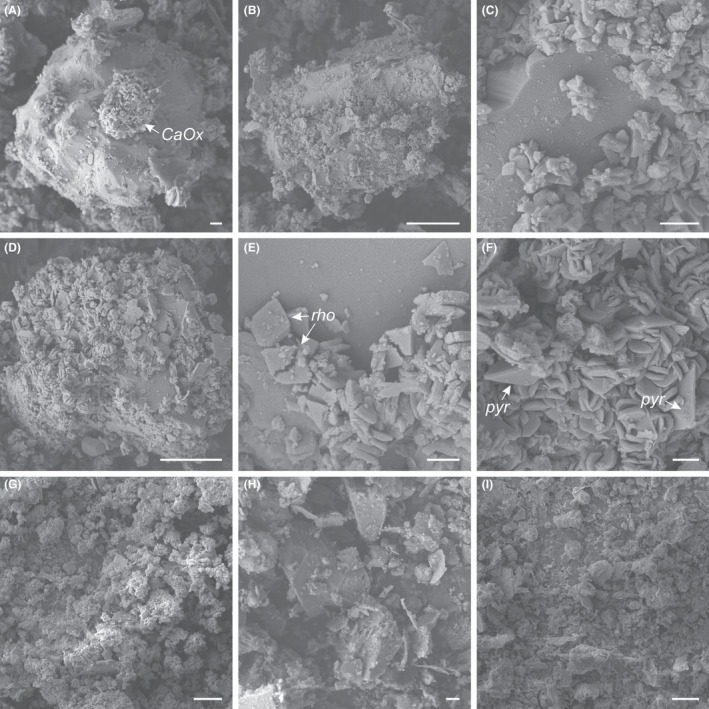
Morphological changes in rock phosphate (RP) particles caused by oxalic acid. SEM images showing calcium oxalate encrustation on RP particles (A–D), morphology of calcium oxalate crystals (E, F), and detail of morphology of treated (G) and untreated RP particles (H, I). Arrows indicate an encrustation of calcium oxalate (CaOx) as confirmed by EDXA (not shown) and rhomboid (*rho*)‐ and bipyramid (*pyr)‐*shaped crystals. Images shown are typical of several images at different oxalic acid doses and reaction times: 50% at 1 h (F), 100% at 15 min (A) and 168 h (G), 150% at 15 min (B, C, E) and 1 h (D). Not all combinations of oxalic acid doses and reaction times are presented due to similar patterns between them. Scale bars: 1 µm (A, C, E, F, H) and 10 µm (B, D, G, I).

SEM images clearly showed the formation of calcium oxalate crystals during the reaction (Fig. 4). In the beginning of the reaction, it was possible to detect the formation of crusts of calcium oxalate crystals on the surfaces of RP particles (Fig. 4A–D). Most of the calcium oxalate crystals possessed a rhomboid shape (Fig. 4E) with some showing a bipyramidal shape (Fig. 4F). Images taken between 1 and 168 h were similar and generally showed a predominance of aggregates of rhomboid crystals.

### RP solubilization with mycogenic oxalic acid

**Fig. 5:**
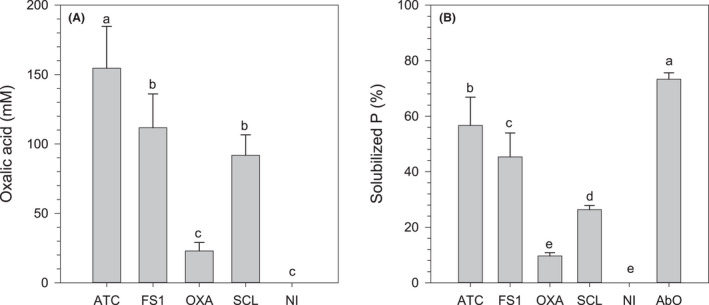
(A) Oxalic acid production by fungi in liquid medium and (B) P solubilized from rock phosphate by applying the culture filtrates or abiotic oxalic acid. ATC: *Aspergillus niger* ATCC 1015, FS1: *A. niger* FS1, OXA: *A. niger ΔoafA* (oxalate overproducer), *Sclerotium rolfsii*, NI: uninoculated control, AbO: abiotic oxalic acid. Treatments sharing a letter are not significantly different (Fisher LSD test, *P* < 0.05). Error bars denote standard deviation (*n* = 3).

*Aspergillus niger* ATCC 1015 produced the highest amount of oxalic acid, reaching 155 mM (Fig. 5A). Biomass‐free spent culture filtrates were applied to solubilize the RP and it was found that maximal P extraction was 57% for the filtrate from *A. niger* ATCC 1015 (Fig. 5B). This value was inferior to that achieved using control abiotic oxalic acid. Moreover, although the amount of oxalic acid applied in the abiotic control was adequate to solubilize 100% of the P contained in the RP, only 73% release was reached.

**Fig. 6:**
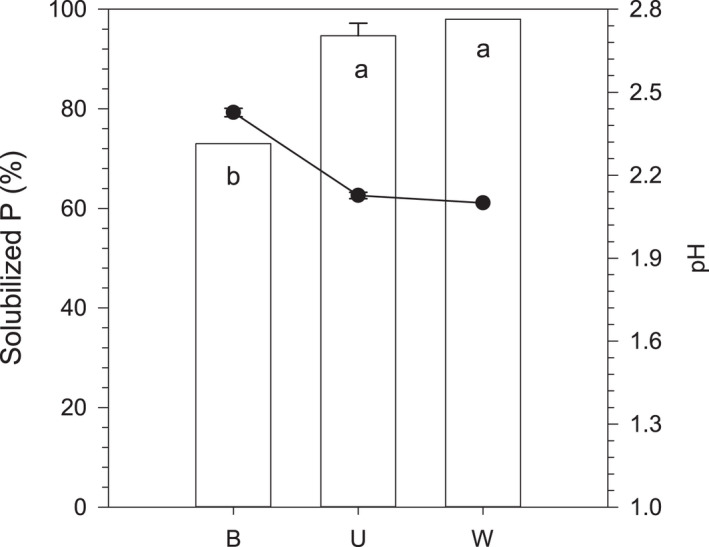
Effect of culture medium buffering on rock phosphate solubilization by oxalic acid. Bars correspond to the solubilized P (% of total P added) and (●) correspond to the pH at the end of the reaction (7 days). Oxalic acid at 100 mM was added to the buffered (B) or unbuffered (U) medium and to Milli‐Q water (W) (control). Treatments sharing a letter are not significantly different (Tukey’s test, *P* < 0.05). Error bars denote standard deviation (*n* = 3).

The culture medium used for oxalic acid production was buffered with 100 mM MES. Without the buffer, nearly 100% of P contained in the RP was solubilized by oxalic acid in the medium (Fig. 6). The initial pH of the buffered medium after addition of 100 mM oxalic acid was 1.71, while the unbuffered medium and Milli‐Q water with the same amount of oxalic acid showed a pH of 1.42. Likewise, at the end of the reaction the pH of the buffered medium was higher than that of the unbuffered medium and Milli‐Q water treatments (Fig. 6).

**Fig. 7:**
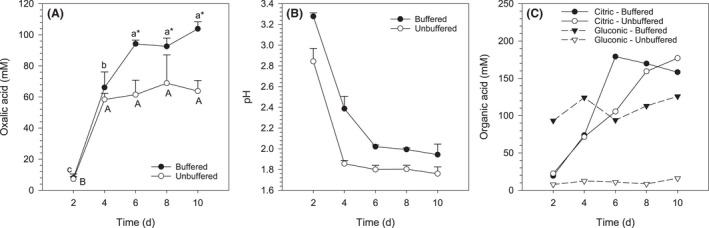
(A) Oxalic acid production by *Aspergillus niger* ATCC 1015 in buffered or unbuffered medium, (B) pH and (C) other organic acids. Time points sharing an uppercase (buffered) or lowercase (unbuffered) letter are not significantly different. *Buffered significantly different from unbuffered (Tukey’s test, *P* < 0.05). Organic acids were not detected in uninoculated controls. Error bars denote standard deviation (*n* = 3)

Oxalic acid production by *A. niger* ATCC 1015 in an unbuffered medium was also examined. The amount of oxalic acid produced in unbuffered medium reach its maximum on the 4th day of incubation and was similar to that of buffered medium (Fig. 7A). Subsequently, oxalic acid production continued only in the buffered medium, reaching a maximum at the 6th day. The pH of the unbuffered medium dropped until the 4th day and stabilized at pH 1.8, while in the buffered medium the pH dropped to 2.0 and stabilized on the 6th day (Fig. 7B). Citric and gluconic acids were produced concomitantly with oxalic acid (Fig. 7C). The effect of medium buffering was more evident for gluconic acid, for which nearly 100 mM was produced in the buffered medium while only traces were detected in the unbuffered medium. Low amounts (less than 10 mM) of malic acid were also detected in both buffered and unbuffered media (not shown).

**Fig. 8:**
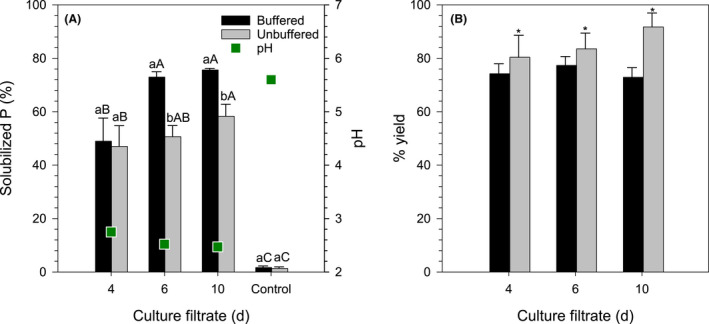
Rock phosphate (RP) solubilization with mycogenic oxalic acid produced by *Aspergillus niger* ATCC 1015 in buffered or unbuffered medium. Culture filtrates from the 4th, 6th and 10th days of incubation (see Fig. 7) and uninoculated controls were mixed with RP at 1.768% (m/v). (A) Solubilized P (% of total P added) and equilibrium pH (green squares). For solubilized P, treatments sharing a letter are not significantly different (Tukey’s test, *P* < 0.05). Lower case letters compare culture filtrates from buffered and unbuffered media within each time/control, and uppercase letters compare different times within each culture filtrate from buffered or unbuffered medium. The equilibrium pH did not differ significantly between buffered and unbuffered media, and therefore, average values are presented. (B) Percentage yield of the reaction based on the amount of oxalic acid present in the culture filtrate (see Fig. 7). *The yield of extracts from unbuffered media was significantly higher (*F* test, *P* < 0.05). Error bars denote standard deviation (*n* = 3).

The application of culture filtrates to solubilize the RP extracted nearly 74% and 55% of the P for the buffered and unbuffered medium respectively (Fig. 8A). Culture filtrates from the 6th and 10th days showed a similar performance, but greater amounts of solubilized P were obtained with culture filtrates from the buffered medium. Conversely, the reaction yield was higher for culture filtrates from the unbuffered medium (Fig. 8B). The equilibrium pH did not differ significantly between buffered and unbuffered media, showing an average value of pH 2.58 for culture filtrates containing mycogenic oxalic acid and pH 5.6 for the uninoculated controls (Fig. 8A).

## Discussion

This research describes a process for RP solubilization employing oxalic acid. Up to 100% P was extracted from RP by oxalic acid when stoichiometric proportions of apatite and oxalic acid (1 Ca_10_(PO_4_)_6_F_2_:10 H_2_C_2_O_4_) were reacted, which means that an excess of oxalic acid was not required to complete the reaction. Our data therefore support the conclusion that the reaction follows a stoichiometry similar to that for RP solubilization by sulfuric acid (Eq. 1), where the oxalate anion plays an analogous role as sulfate, forming insoluble calcium oxalate instead of calcium sulfate (Eq. 2). As predicted by Eq. 2, XRD data showed that while the apatite was consumed in the reaction, most of the Ca released was precipitated by oxalate producing mainly whewellite (CaC_2_O_4_·H_2_O), a very stable monohydrate calcium oxalate, and traces of weddellite (CaC_2_O_4_·2H_2_O), a dihydrate calcium oxalate. Accordingly, SEM images revealed a predominance of rhomboidal‐ over bipyramid‐shaped crystals, which are typical morphologies of whewellite and weddellite respectively (Hartl *et al.,* 2007). Both minerals are found in natural environments as a result of microbial, plant or animal metabolism, but whewellite generally predominates due to its higher stability (Gadd, 1999; Gadd *et al.,* 2014; Ruiz‐Agudo *et al.,* 2017; Mendes *et al.,* 2020a). In soil, calcium oxalate participates in Ca and P cycles, but rarely ever accumulates due to microbial degradation (Morris and Allen, 1994; Dauer and Perakis, 2014). Therefore, it is likely that application of the reaction product to soil without further processing to remove the calcium oxalate could be effective as a P fertilizer as well as a Ca source. This would represent an advantage over the conventional process employing sulfuric acid, in which 4.5–5.5 t of gypsum is obtained as by‐product during the production of 1 t P_2_O_5_ (Schrödter *et al.,* 2000; Gilmour, 2014). Moreover, oxalic acid forms sparingly soluble oxalates with many potentially toxic metals that may be present in RP, such as Cu, Ni and Pb (Lide, 2004; Fomina *et al.,* 2005, 2010; O'Neil, 2013; Liang and Gadd, 2017), which may reduce the risk of soil contamination. Potential Al toxicity is more evident in very acidic soil types but it should be noted that rock phosphate application is subject to controls regarding composition and if potentially toxic elements exceed permissible values then it should not be used without some beneficiation process.

The main factor driving the reaction kinetics was the concentration of oxalic acid. When oxalic acid was the limiting reactant, the reaction’s theoretical yield was reached in less than 24 h. However, oxalic acid in excess or at 100% of the required dose retarded the reaction due to calcium oxalate encrustation on RP surfaces. SEM images revealed partial encrustation of the RP particles after 15 min of reaction, and the particles were almost completely covered after 1 h. Likewise, calcium sulfate encrustation can impair RP solubilization when excess sulfuric acid is applied (Hatfield, 1964; Schrödter *et al.,* 2000; Gilmour, 2014). However, over a wider time frame the effect of encrustation was overcome so that the reaction reached completion after 168 h for both 100% and 150% of the required dose of oxalic acid. Therefore, the reaction reached a 100% yield using stoichiometric proportions of the reactants, which is a significant advantage over the conventional wet process used in phosphoric acid plants, which require excess sulfuric acid (2–3% SO_4_ excess) (Gilmour, 2014). Oxalic acid has been reported as being more efficient than sulfuric acid to solubilize RP due to its capacity to form stable complexes with Ca (Kpomblekou‐A and Tabatabai, 1994, Kpomblekou‐A and Tabatabai, 2003; Mendes *et al.,* 2020b)). The solubility of calcium oxalate (*K*_sp_ 2.32 × 10^−9^) is 2 × 10^4^ times lower than that of CaSO_4_ (Lide, 2004). Therefore, the oxalate anion acts as a much stronger Ca sink in the solubilization reaction (Robinson *et al.,* 1992). Moreover, calcium oxalate is soluble only at extremely acidic conditions (pH < 2) (Lide, 2004; Mendes *et al.,* 2014). Therefore, at the pH values measured in this study, most of the Ca released from apatite formed insoluble calcium oxalate.

In fact, our data show that the Ca released from apatite was promptly precipitated by oxalate, forming the sparingly soluble minerals whewellite and weddellite. XRD data showed that the onset of whewellite accumulation was detected earlier than 15 min. A previous report showed that calcium oxalate precipitation commenced 760 s after Ca and oxalate ions were reacted in solution (Ruiz‐Agudo *et al.,* 2017).

The other reactant, the RP, had a minor effect on the reaction. An increase in the proportion of solids (i.e. RP dose) decreased the percentage of P solubilized from RP, as described for other bioleaching systems (Chi *et al.,* 2006; Xiao *et al.,* 2008; Mendes *et al.,* 2013; Calle‐Castañeda *et al.,* 2018). The reason for this is the limitation of the other reactant, *i.e*. oxalic acid. As oxalic acid was applied at a fixed concentration (100 mM), it was the limiting reactant when 2.5%, 5% and 10% solids were used. In these treatments, the percentage yield of the reaction was 100%, indicating that all the available oxalic acid reacted with the RP. Since the RP contained a significant number of mineral contaminants, this result highlights the efficiency and specificity of oxalic acid to react with apatite ores, suggesting that little or no oxalic acid was consumed by side reactions. It is worth mentioning that the RP used was not treated by any of the beneficiation steps applied in industry to remove contaminants, such as washing, flotation and calcination (Al‐Fariss *et al.,* 1992; Gilmour, 2014).

Some differences were found when comparing abiotic and biologically produced oxalic acid. Although *A. niger* ATCC 1015 produced amounts of oxalic acid equivalent to those used in the abiotic assays, the reaction between biomass‐free spent culture filtrates with RP did not reach 100% of the theoretical yield. The main reason for this appeared to be the buffer used in the medium to increase oxalic acid production by *A. niger*, which consumed a proportion of the protons that would have reacted with the RP (Robinson *et al.,* 1992). Oxalic acid production by *A. niger* is favoured at pH 6 (Kubicek *et al.,* 1988; Strasser *et al.,* 1994; Ruijter *et al.,* 1999). At a low medium pH, oxalic acid production decreases and can be completely inhibited when the pH is lower than 2 (Kubicek *et al.,* 1988; Ruijter *et al.,* 1999). Our data show that medium buffering increased oxalic acid production by 67% because the medium pH remained higher than 2 until the 6th day, while in unbuffered medium the pH dropped below 2 on the 4th day. Application of these culture filtrates to solubilize RP resulted in higher P extraction by the media with more oxalic acid, whether derived from buffered or unbuffered medium. Therefore, a higher oxalic acid production can offset the partial consumption of protons by the buffer. It should be mentioned that other organic acids besides oxalic were produced through growth, but their contribution to RP solubilization seemed to be minimal. Surprisingly, the oxalate overproducing *A. niger* mutant (*ΔoafA*) (Poulsen *et al.,* 2012) did not perform well at the conditions used in this study. Poulsen *et al*. (2012) used a cultivation medium with a glucose concentration of 20 g l^−1^ glucose. When glucose was exhausted, the mutant was more efficient than wild‐type *A. niger* ATCC1015 in converting previously produced gluconate into oxalic acid. Since the medium used in this study contained an optimal sugar concentration (100 g l^−1^ sucrose) for oxalic acid secretion (Strasser *et al.,* 1994), the advantage of the mutant over the wild type was probably nullified. Moreover, the use of sucrose instead of glucose could decrease the rate of gluconate production by the mutant and, therefore, the conversion of gluconate to oxalic acid. Although the performance of mycogenic oxalic acid for RP solubilization was lower than that obtained with abiotic oxalic acid, the reaction still extracted 74% of the P contained in the RP. Another heterotrophic system using biogenic gluconic acid to solubilize RP extracted 60% P (Goldstein *et al.,* 1993). Phosphoric acid plants usually recover 85%–98% of the P depending on RP quality and plant management (Gilmour, 2014). Bearing in mind that mycogenic oxalic acid relies on a renewable carbon and energy source, while sulfuric acid is produced from elemental sulfur (Gilmour, 2014; Calle‐Castañeda *et al.,* 2018; Mendes *et al.,* 2020b), that difference could be compensated for. RP solubilization systems based on microbial chemolithotrophic oxidation of elemental sulfur have also been investigated (Chi *et al.,* 2006; Calle‐Castañeda *et al.,* 2018). Calle‐Castañeda *et al*. (2018) reported a P extraction efficiency of 94% using biogenic sulfuric acid produced by the chemolithotrophic bacterium *Acidithiobacillus thiooxidans*. However, the culture medium used to produce the acid required previous acidification and the biogenic acid was responsible for just 40% of the total P extracted. Moreover, like the conventional sulfuric acid production route, this system relies on provision of elemental sulfur. Furthermore, chemolithotrophic metabolism is slower than heterotrophic metabolism, often taking weeks to reach adequate acid concentrations (Calle‐Castañeda *et al.,* 2018).

In summary, this research highlights the potential of oxalic acid as an efficient agent to extract P from RP. The reaction of oxalic acid with apatite is rapid and efficient, reaching completion with stoichiometric proportions of the reactants. We have also demonstrated that mycogenic oxalic acid can efficiently extract P from RP. Thus, this work provides insights for a potential bioleaching scheme for RP solubilization employing biogenic oxalic acid produced from renewable substrates. A biotechnological route for RP solubilization based on mycogenic oxalic acid could feasibly make use of low‐grade RPs to produce phosphate fertilizer and allow the incorporation of currently rejected ores into production. Moreover, alternative P sources, such as animal bone char (Vassilev *et al*., 2013, 2013,2013, 2013) and struvite from wastewater treatment plants (Suyamud *et al.,* 2020), could be processed in such a biotechnological scheme for recycling P. This would represent a significant advance towards the sustainability of P resources.

## Experimental procedures

### Rock phosphate

Rock phosphate (RP) was supplied by Morro Verde Mineração, Pratápolis, Minas Gerais state, Brazil. The rock was ground in a ball mill and serially sieved to pass through 125 and 63 µm sieves. A preliminary assessment revealed that the < 63 µm fraction allowed better solubilization (not shown), and this granulometry was used throughout the experiments. The elemental and mineralogical composition of the RP was determined by X‐ray fluorescence spectroscopy (XRF) and powder X‐ray diffraction (XRD) respectively.

### Organisms and media

*Aspergillus niger* ATCC 1015 and *A. niger ΔoafA* (an oxalate overproducer) (Poulsen *et al.,* 2012) were used. *Aspergillus niger* FS1 and *Sclerotium rolfsii* were obtained from the LAMIF culture collection (Universidade Federal de Uberlândia, Monte Carmelo, MG, Brazil). These fungi are known oxalic acid producers (Punja and Jenkins, 1984; Poulsen *et al.,* 2012; Gadd *et al.,* 2014; Mendes *et al.,* 2014). All fungi were maintained on malt extract agar (MEA; Sigma‐Aldrich, St. Louis, MO, USA) at 25°C in the dark.

Batch cultures were performed using a sucrose medium optimized for fungal oxalic acid production (Strasser *et al.,* 1994), containing (g l^−1^ Milli‐Q water): sucrose 100, NaNO_3_ 1.5, KH_2_PO_4_ 0.5, MgSO_4_·7H_2_O 0.025, KCl 0.025 and yeast extract 1.6. The pH was adjusted to 6 using 0.1 M NaOH and, unless otherwise stated, buffered with 0.1 M MES. The medium (100 ml) was transferred to 250 ml Erlenmeyer flasks and autoclaved at 121°C for 20 min.

### Reaction parameters of RP solubilization by oxalic acid

Two experiments using pure oxalic acid dihydrate (Sigma‐Aldrich, St. Louis, MO, USA) were performed to establish the main parameters of the RP solubilization reaction. The reactions were carried out at room temperature (~20°C) in 50 ml conical centrifuge tubes. The tubes were filled with 25 ml of an oxalic acid solution or Milli‐Q water (controls) and incubated at 50 r.p.m. on a roller mixer. Based on the fungal capacity to produce oxalic acid, the experiments were performed using a 100 mM oxalic acid solution which had an initial pH of 1.34.

The first experiment aimed at determining the reaction time and the effect of the proportion of solids, *i.e*. the RP dose. RP was applied at 1, 2.5, 5 and 10% (m/v) to the 100 mM oxalic acid solution and incubated as described. Samples of the reaction medium were taken at 15, 30, 60 and 120 min, centrifuged at 3200 *g* for 10 min to remove any remaining particles and the supernatants analysed for soluble phosphate (Pi). Experiments were carried out in triplicate.

The second experiment examined the oxalic acid dose and the reaction time over a wider time frame (0.25, 0.5, 1, 24, 72, 120 and 168 h). Oxalic acid was applied at 50%, 100% and 150% of the required dose to extract all the P contained in the RP. The tubes were incubated in triplicate as described and samples were collected at each time point. These were centrifuged at 3200 *g* for 10 min, and the supernatant analysed for pH and soluble inorganic phosphate (Pi) and Ca. The sediment was dried in a desiccator with silica gel for at least one week and analysed by powder XRD, scanning electron microscopy (SEM) and energy‐dispersive X‐ray analysis (EDXA).

To determine the amounts of the reactants (oxalic acid and RP), it was assumed that oxalic acid reacts with the apatite in a similar manner as sulfuric acid (Gilmour, 2014):

(1) $$\begin{array}{ll} & {\text{Ca}}_{10}{\left({\text{PO}}_{4}\right)}_{6}F_{2}+10H_{2}{\text{SO}}_{4}+x\\ & H_{2}O\to 10{\text{CaSO}}_{4}\cdot {\left(H_{2}O\right)}_{x}+6H_{3}{\text{PO}}_{4}+2\text{HF} \end{array}$$

(2) $$\begin{array}{ll} & {\text{Ca}}_{10}{\left({\text{PO}}_{4}\right)}_{6}F_{2}+10H_{2}C_{2}O_{4}+x\\ & H_{2}O\to 10{\text{CaC}}_{2}O_{4}\cdot {\left(H_{2}O\right)}_{x}+6H_{3}{\text{PO}}_{4}+2\text{HF} \end{array}$$

Therefore, all calculations for the required doses of oxalic acid and RP were carried out based on the stoichiometric relationship between apatite and oxalic acid (Eq. 2). In the second experiment, the oxalic acid concentration was fixed (100 mM) and the amount of RP was varied: 884.1, 442.1 and 294.7 mg RP per tube for the 50, 100 and 150% oxalic acid dose respectively. To rule out possible effects from the different proportions of solids applied, an additional test employed a fixed RP amount [5% (m/v)] while varying the oxalic acid concentration accordingly. Both experimental set‐ups returned the same results (not shown).

### RP solubilization with mycogenic oxalic acid

Initially, four fungi (*A. niger* ATCC 1015, *A. niger ΔoafA*, *A. niger* FS1 and *S. rolfsii*) were screened for their capacity for producing oxalic acid. Three mycelial plugs of each fungus were inoculated in a 250 ml Erlenmeyer flask containing 50 ml of sterile sucrose medium. Inoculated flasks were incubated in an orbital shaker at 250 r.p.m. and 25°C in the dark for 7 days. Uninoculated flasks were used as controls. At the end of the incubation period, the spent medium was filtered through a paper filter (Whatman Grade 4) to remove fungal biomass and then vacuum filtered through a 0.45 µm pore size cellulose nitrate membrane to remove any remaining particulate material and fungal biomass. Oxalic acid in the filtrate was measured by high‐performance liquid chromatography (HPLC). To assess RP solubilization by the biomass‐free spent culture medium, 10 ml of the culture filtrates was mixed with 270.7 mg RP in a 50 ml conical centrifuge tube and incubated for 5 days at room temperature on a roller mixer at 50 r.p.m. The amount of RP used corresponded to the stoichiometric proportion (Eq. 2) which would fully react with the highest concentration of oxalic acid detected in the culture filtrates (155 mM). After the incubation period, the reaction medium was centrifuged at 3200 *g* for 10 min and analysed for soluble Pi.

An evaluation of the effect of culture medium buffering on RP solubilization was carried out with abiotic oxalic acid. For this, oxalic acid was added to a 100 mM final concentration, to 25 ml of buffered (0.1 M MES) or unbuffered sucrose medium and to Milli‐Q water (control). The media were prepared as described previously and sterilized before oxalic acid addition. RP was added to the required stoichiometric proportion to allow full solubilization, i.e. 442 mg per tube. The reaction was carried out for 7 days in 50 ml conical centrifuge tubes incubated at room temperature on a roller mixer at 50 r.p.m. After the incubation period, the reaction medium was centrifuged (3200 *g*, 10 min) and the supernatant analysed for soluble Pi and pH.

Since *A. niger* ATCC 1015 proved to be most effective in the screening for oxalic acid production, a further experiment was carried out with this organism to evaluate oxalic acid production in buffered (0.1 M MES) and unbuffered media and the efficiency of the culture filtrates for RP solubilization. Media were prepared as described previously, with 75 ml being added to 250 ml Erlenmeyer flasks. Each flask was inoculated with 10^7^ conidia from a conidial suspension prepared in 0.1 % (v/v) Tween 80. Uninoculated flasks were used as controls. The flasks were incubated in an orbital shaker at 250 r.p.m. and 30°C in the dark for 10 days, and samples collected every 2 days. The spent media were vacuum filtered through 0.45 µm pore size cellulose nitrate membrane filters and analysed for oxalic acid (and other organic acids) and pH. After this, 25 ml of the culture filtrates from the 4th, 6th and 10th days of reaction was mixed with 442 mg RP in a 50 ml conical centrifuge tube and incubated for 7 days at room temperature on a roller mixer at 50 r.p.m. The amount of RP used corresponded to the stoichiometric proportion (Eq. 2) to fully react with the highest concentration of oxalic acid detected in the culture filtrates (100 mM). At the end of the experiment, the reaction medium was centrifuged (3200 *g*, 10 min) and the supernatant analysed for soluble Pi.

### Analytical procedures

Soluble Pi in the reaction medium was determined by the spectrophotometric molybdenum blue method (Murphy and Riley, 1962). For all experiments using the sucrose medium as reaction medium, the amount of soluble Pi added to the medium (0.5 g l^−1^ KH_2_PO_4_) was subtracted from the measured concentration. To allow comparison between experiments with different RP doses, data were presented as a percentage of P solubilized from the RP, calculated as the ratio between the amount of solubilized P and the total P added. The percentage yield of the reaction was calculated using the formula:

(3) $$\%\;\text{yield}=\frac{\text{actual}\;\text{yield}}{\text{theoretical}\;\text{yield}}\times 100$$

where the theoretical yield is the maximum amount of solubilized P achievable calculated with respect to the limiting reactant (RP or oxalic acid).

Soluble Ca was measured by atomic absorption spectrophotometry (AAS) using an AAnalyst 400 spectrometer (PerkinElmer Instruments, Waltham, MA, USA). The percentage of Ca precipitated as calcium oxalate was estimated as follows:

(4) $$\text{Precipitated Ca}\;\left(\%\right)=\frac{\text{Ca}\;\text{released}‐\text{Soluble}\;\text{Ca}}{\text{Ca}\;\text{released}}\times 100$$

where the Ca released was calculated with respect to the amount of solubilized P following the reaction stoichiometry, i.e. 10 mol Ca is released from apatite for each 6 mol P solubilized (Eq. 2).

Oxalic acid and other organic acids were determined by HPLC using an Ultimate 3000 HPLC System (Dionex, Sunnyvale, CA, USA) equipped with a variable wavelength detector (VWD). Chromatographic separations were performed using an Aminex HPX‐87H Column (300 mm × 7.8 mm, Bio‐Rad Laboratories, Hercules, CA, USA) with the following conditions: sample injection volume, 20 µl; mobile phase, 4 mM H_2_SO_4_; flow rate, 0.6 ml min^−1^; column temperature, 35°C; and analysis time, 18 min. Compounds were detected at 210 nm and quantified based on standard chromatograms for pure solutions of oxalic, citric, gluconic, malic, fumaric, itaconic and succinic acid.

For morphological examination, dried sediment samples were mounted on aluminium stubs using carbon adhesive tape and coated with a 10 nm layer of gold and palladium using a Cressington 208HR Sputter Coater (Cressington Scientific Instruments, Watford, UK). Samples were examined using a JEOL JSM‐7400F Field Emission Scanning Electron Microscope (JEOL Ltd., Tokyo, Japan) operating at an accelerating voltage of 5 kV. Elemental composition of selected crystals was determined using an embedded energy‐dispersive X‐ray analysis system (Oxford Instruments Inca, Abingdon, Oxfordshire, UK), operating at an accelerating voltage of 20 kV.

The mineralogical composition of the powdered materials was estimated using Rietveld refinement techniques applied to powder X‐ray diffraction (XRD) traces. Rietveld refinement is primarily a technique for the refinement of crystal structure parameters from powder X‐ray diffraction data. However, a secondary application of the technique is the quantitative analysis of mixtures of crystalline phases, which allows analysis with a relatively high degree of accuracy (error < 1.0%) (Bish and Howard, 1988). XRD was conducted using a Siemens D5000 Powder X‐ray Diffractometer with a Cu‐Kα source operating at 40 mA and 40 kV. The traces were obtained at a rate of 0.33° 2*θ*/min in angular increments of 0.1° 2*θ*. Where Rietveld refinement is used on materials containing a non‐crystalline component, an internal standard can be added to allow quantitative measurement of the total amorphous content. However, preliminary assessment using a corundum internal standard indicated that the amorphous content of the materials was negligible, and therefore, the traces obtained for Rietveld refinement analysis were run without internal standards. Rietveld refinement was conducted using the MAUD software package (Lutterotti, 2010).

The chemical composition of RP was determined using X‐ray fluorescence spectroscopy. Samples were prepared as pressed powder pellets which were analysed using a Panalytical Zetium 2.4W X‐ray fluorescence spectrometer (Malvern Panalytical, Malvern, UK).

### Statistics

Experiments were performed using a completely randomized design with three replicates. Data were subjected to ANOVA, and treatments were compared using Tukey’s or Fisher’s LSD tests (*P* < 0.05).

## Conflict of interest

The authors declare no conflicts of interest.
